# A Comprehensive Genome Survey Provides Novel Insights into Bile Salt Hydrolase (BSH) in *Lactobacillaceae*

**DOI:** 10.3390/molecules23051157

**Published:** 2018-05-11

**Authors:** Lifeng Liang, Yunhai Yi, Yunyun Lv, Junwei Qian, Xuejing Lei, Gengyun Zhang

**Affiliations:** 1 BGI Education Center, University of Chinese Academy of Sciences, Shenzhen 518083, China; lianglifeng@genomics.cn (L.L.); yiyunhai@genomics.cn (Y.Y.); lvyunyun@genomics.cn (Y.L.); 2 State Key Laboratory of Agricultural Genomics, BGI-Shenzhen, Shenzhen 518083, China; qianjunwei@genomics.cn (J.Q.); leixuejing@genomics.cn (X.L.); 3 Shenzhen Key Lab of Marine Genomics, Guangdong Provincial Key Lab of Molecular Breeding in Marine Economic Animals, BGI Academy of Marine Sciences, BGI Marine, BGI, Shenzhen 518083, China; 4 Key Lab of Genomics, Chinese Ministry of Agriculture, BGI-Shenzhen, Shenzhen 518083, China

**Keywords:** bile salt hydrolase (BSH), *Lactobacillaceae*, genome survey, BSH classification, cholesterol-lowering probiotics

## Abstract

Bile salt hydrolase (BSH) is a well-known enzyme that has been commonly characterized in probiotic bacteria, as it has cholesterol-lowering effects. However, its molecular investigations are scarce. Here, we build a local database of BSH sequences from *Lactobacillaceae* (BSH–SDL), and phylogenetic analysis and homology searches were employed to elucidate their comparability and distinctiveness among species. Evolutionary study demonstrates that BSH sequences in BSH–SDL are divided into five groups, named BSH A, B, C, D and E here, which can be the genetic basis for BSH classification and nomenclature. Sequence analysis suggests the differences between BSH-active and BSH-inactive proteins clearly, especially on site 82. In addition, a total of 551 BSHs from 107 species are identified from 451 genomes of 158 *Lactobacillaceae* species. Interestingly, those bacteria carrying various copies of BSH A or B can be predicted to be potential cholesterol-lowering probiotics, based on the results of phylogenetic analysis and the subtypes that those previously reported BSH-active probiotics possess. In summary, this study elaborates the molecular basis of BSH in *Lactobacillaceae* systematically, and provides a novel methodology as well as a consistent standard for the identification of the BSH subtype. We believe that high-throughput screening can be efficiently applied to the selection of promising candidate BSH-active probiotics, which will advance the development of healthcare products in cholesterol metabolism.

## 1. Introduction

Currently, cardiovascular disease (CVD) is one of the main causes of health problems and mortality worldwide [1]. Although multiple risk factors could give rise to CVD, elevated serum cholesterol is the major cause correlating to the progression of CVD in humans. Numerous epidemiological and clinical studies observed that the high serum level of low-density lipoprotein cholesterol (LDL-C) and total cholesterol (TC) were strongly associated with the incidence of CVD [2]. Cholesterol in the human body is absorbed from food in the small intestine or de-novo synthesized in the liver. Cholesterol is broken down into the primary bile salts cholic acid (CA) or chenodeoxycholic acid (CDCA) under enzymolysis by more than 17 hepatic enzymes. CA is subsequently combined with taurine or glycine, to form taurocholic acid (TCA) and glycocholic acid (GCA), while CDCA utilizes it to synthesize taurochenodeoxycholic acid (TCDCA) and glycochenodeoxycholic acid (GCDCA) [3,4]. The two groups within each macromolecular compound are conjugated. In addition, these four compounds are then accumulated in the body and have been reported to cause adverse effects, including cytotoxicity [5], severe cancer-promoting consequences [6], colonic and oesophageal carcinogenesis in the context of obesity [7,8], and even liver tumour development [9].

In the intestine, TCA, GCA, TCDCA and GCDCA can be decomposed into unconjugated bile acids (CA, CDCA) and amino acid residues via hydrolysis by bile salt hydrolase (BSH, EC 3.5.1.24), which is mainly derived from *Bacteroides*, *Clostridium*, *Lactobacillus*, *Bifidobacterium* and *Listeria*. A small proportion of CA and CDCA will be 7α-dehydroxylated into secondary bile salts (DCA and LCA) by the actions of *Clostridium* and *Eubacterium* [10]. Farnesoid X receptor (FXR) and G-protein-coupled bile salt receptor 1 (GPBAR1, also known as TGR5) are then stimulated by CA, CDCA, DCA or LCA, in order to regulate host metabolism, such as increasing the cholesterol efflux and limiting the bile salt synthesis in hepatocytes. The majority of CA and CDCA are reabsorbed in the intestinal lumen, and the rest are excreted into feces. Thus, the presence of gastrointestinal bacteria carrying active BSH could accelerate cholesterol metabolism and reduce serum cholesterol levels, thus lowering the risk of CVD [11,12,13,14,15]. Previous studies have indicated that hypercholesterolemic adults treated with certain probiotics, such as *Lactobacillus reuteri* NCIMB 30242 [16], *Lactobacillus acidophilus* La5 [17], and *Lactobacillus plantarum* CECT 7527, 7528 and 7529 [18], could have lower serum TC and LDL-C levels than before. Interestingly, most of these probiotics were proven to be BSH-active. Therefore, BSH is an important enzyme that can affect the regulation of host serum cholesterol metabolism.

BSH is a cytoplasmic enzyme belonging to the N-terminal nucleophile (NTN-) hydrolase superfamily. Variable numbers of *bsh* genes exist in most *Lactobacillaceae* species (Table S1), and their function is divergent. For instance, most *Lactobacillus plantarum* carrying four *bsh* genes, such as WCFS1 [19] and ST-III [20], were not able to hydrolyze bile salts (BAs) with high efficiency. It has been proposed that penicillin acylase could contribute to the consumption of BAs, as it is also a member of the NTN-hydrolase superfamily [21]. Another study demonstrated that two BSHs had different substrate specificities in *Lactobacillus acidophilus* NCFM [22]. In addition, only one of the two *bsh* genes in *Lactobacillus fermentum* MTCC 8711 performed BSH activity [23]. Moreover, many strains carrying multiple *bsh* gene copies have been reported to be bile resistant, but the responsible genes have not been identified. Thus, it is challenging to find out the exact BSH enzymes that could hydrolyze BAs efficiently.

Although several *bsh* gene-sequence analyses have been reported in common probiotics, the *bsh* gene distribution, evolutionary relationship, and the correlations between sequence characteristics and BA hydrolyzation activity in *Lactobacillaceae* are still unclear. With the rapid increase in available genome data of *Lactobacillaceae*, it is possible and imperative to further explore *bsh* gene biodiversity. In this study, a BSH sequence seed database was built to probe into the characteristics of BSH-active sequences both in their evolutionary relationship and active sites, revealing a reliable model for BSH classification. In addition, a large-scale genome survey in *Lactobacillaceae* enhances our recognition of BSH-active probiotics. Taken together, this information provides novel insights into *bsh* in *Lactobacillaceae*, and a high-throughput approach to select potential cholesterol-lowering probiotics.

## 2. Results

### 2.1. Establishment of BSH Database in Lactobacillaceae

A total of 60 bile salt hydrolase protein sequences in 34 *Lactobacillaceae* species were collected from the Kyoto Encyclopedia of Genes and Genomes (KEGG) database to build a bile salt hydrolase seed database (BSH–SDL) (Table S1). Every BSH protein sequence was selected from the representative strain in each species, except that two strains were selected from *Lactobacillus johnsonii*.

**Figure 1:**
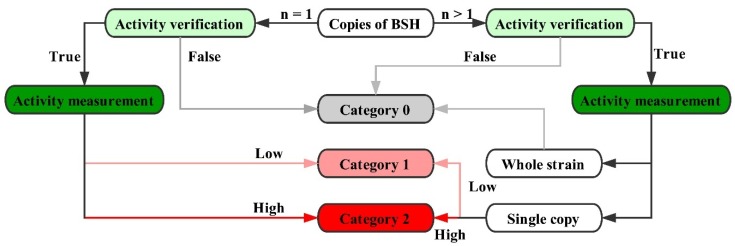
Scoring rules of BSH.

All these sequences were divided into three categories based on their level of biological activation in hydrolyzing BAs, according to previous studies. As shown in Figure 1, if a strain carries multiple *bsh* gene copies, the sequences with high BSH activity that have been confirmed by previous studies were graded as “2”, while those with low BSH activity were “1”. If a BSH-active strain was found to have only one *bsh* gene in the genome, this BSH was noted as “2”. If the *bsh* genes and BSH activity in a strain have not been verified, all BSHs of this strain were noted as “0”. If the relevant experiments for a strain have not been reported, but several *bsh*-homologous genes exist in the genome, these *bsh* genes were still marked as “0”. All the scoring numbers are shown in the “Category” column of Table S1. Accurate data collection and complete information for each strain are the fundamental basis of our analysis.

Four *bsh* genes (lp_0067, lp_2572, lp_3362 and lp_3536) were found in *Lactobacillus plantarum* WCFS1, and their BSH activities have been verified by molecular cloning experiments. Those results have shown that lp_0067 expressed a BSH which had the highest BSH activity, while other *bsh* genes had lower activity. Thus, lp_0067 was graded as “2”, while other *bsh* genes were noted as “1” [19]. The *Pediococcus acidilactici* genome contains only one *bsh* gene, and its BSH activity has been reported [24], so this BSH was noted as “2”. *Lactobacillus fermentum* was proven to have cholesterol-lowering effects and several *bsh* gene copies were identified; however, the detailed information of functional properties about each *bsh* gene was not reported. In this case, all *bsh* genes of *Lactobacillus fermentum* were marked with “0” [23]. Although *Pediococcus damnosus* possesses one *bsh* gene, related BSH activity properties have not been reported yet, so it was noted as “0”. Therefore, there are 18 sequences with high BSH activity, four with lower BSH activity, and 38 without BSH-active records in total in BSH–SDL.

### 2.2. Phylogenetic Analysis

**Figure 2:**
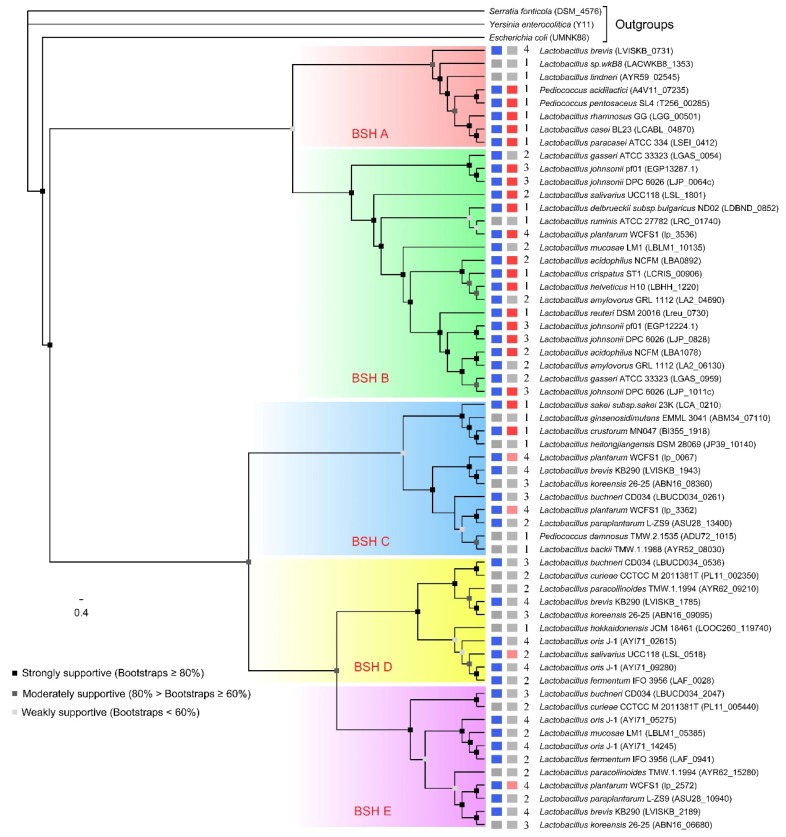
Phylogenetic tree of BSHs in 34 *Lactobacillaceae* species. The BSH subtypes are shaded by various colors. BSH A, B, C, D and E are shown in red, green, blue, yellow and purple, respectively. On the right-hand side, the rectangle of the first column denotes experimental reports of this strain or *bsh* genes; blue means it has been reported before and grey means it has not. The second rectangle denotes the scoring type of this BSH (see more details in Table S1), categories 2, 1 and 0 are marked with red, light red and grey, respectively. The number beside it indicates the copy number of the *bsh* gene in this strain.

Phylogenetic analysis was employed to reveal the evolutionary relationship among BSH proteins in BSH–SDL. Five distinct clades obviously emerged from the phylogenetic tree (Figure 2). It is interesting to note that almost all BSHs with high activity are gathered in two clades. Therefore, five subtypes of BSH are presumed to exist in *Lactobacillaceae* species based on the results, and here we named them “BSH A”, “BSH B”, “BSH C”, “BSH D” and “BSH E”. Most BSH A and BSH B proteins have been reported to exhibit greater BSH activity according to previous studies, while the majority of BSH C, BSH D and BSH E proteins have not been verified to be BSH-active yet.

There are eight proteins classified in the BSH A subtype, including five BSH sequences verified in *Pediococcus* (*Pediococcus acidilactici* and *Pediococcus pentosaceus*), *Lactobacillus casei* group (*Lactobacillus casei* and *Lactobacillus paracasei*), *Lactobacillus rhamnosus*, and three BSHs with unknown activity, which were predicted from *Lactobacillus brevis*, *Lactobacillus lindneri* and *Lactobacillus* sp. wkB8. There are 18 BSH proteins from 12 species in the BSH B subtype, and they have been identified in commonly applied probiotics, including *Lactobacillus plantarum*, *Lactobacillus gasseri*, *Lactobacillus johnsonii*, *Lactobacillus salivarius*, *Lactobacillus reuteri*, *Lactobacillus acidophilus* and *Lactobacillus delbrueckii* subsp. *bulgaricus*, *Lactobacillus mucosae*, *Lactobacillus amylovorus*, and *Lactobacillus helveticus*, except an unproven BSH in *Lactobacillus ruminis* (Figure 2).

Two proteins with high BSH activity from *Lactobacillus sakei* and *Lactobacillus crustorum* were assigned to the BSH C subtype group, while the other proteins in this group have not demonstrated high BSH activity. The majority of proteins in BSH C, BSH D and BSH E were predicted to be bile salt hydrolases but without experimental substantiation. They mainly come from species with multiple BSH copies, except for four BSHs with relatively lower activity in *Lactobacillus plantarum* and *Lactobacillus salivarius*. Moreover, a strain with multiple BSH copies has diverse BSH subtypes. For instance, four BSHs of *Lactobacillus plantarum* are classified into three BSH subtypes, BSH B, BSH C and BSH E, and four BSH copies of *Lactobacillus brevis* were distributed in BSH A, BSH C, BSH D and BSH E subtypes.

### 2.3. Sequence Analysis of BSH

**Figure 3:**
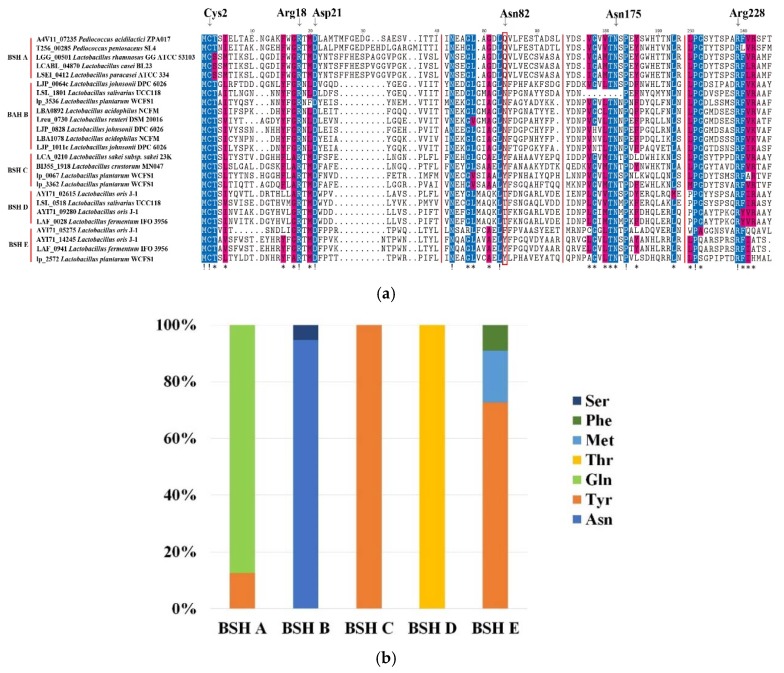
Analysis of catalytic residues of BSH proteins. (**a**) Multiple-sequence alignment of five BSH subtypes in representative species. Six catalytic residues are marked by top arrow, and the site 82 is highlighted by the red box. The residues with sequence identity >50% and >80% were shaded by blue and yellow blocks, respectively; “!” and “*” were noted in the bottom side which residues identity =100% and >80%, respectively. (**b**) amino acid statistics of site 82 in six BSH subtypes from all BSH–SDL sequences (see more details of other sites in Table S2).

Previous study has identified six catalytic residues as active sites of BSH, including Cys2, Arg18, Asp21, Asn82, Asn175 and Arg228 [25]. Multiple-sequence alignment of BSH protein sequences in BSH–SDL showed that Cys2, Arg18, Asp21, Asn175 and Arg228 were highly conserved among all BSH subtypes, whereas Cys2, Arg18 and Asp21 were absent in few BSH A sequences (Figure 3a, Table S2). The site 82 was highly diverse in comparison to five other sites, including the Ser, Phe, Met, Thr, Gln, Tyr and Asn types (Figure 3b). Gln82 and Asn82 were commonly seen in this catalytic site of BSH A and BSH B, accounting for 87.5% and 94.74% in all protein sequences, respectively. In addition, the site 82 of BSH C and BSH D were all Tyr82 and Thr82, respectively, while BSH E possessed three types, including Tyr82 (72.73%), Met82 (18.18%) and Phe82 (9.09%) (Table S2). It was indicated that the site 82 was closely related to the activity of BSH, and Gln82 and Asn82 were assumed to be essential requirements for BSH with high hydrolase activity. Furthermore, the residues near catalytic sites were rather conserved, while the other sites were not. It is demonstrated that although BSH protein sequences have low homology, some of them still have a similar ability to perform hydrolytic reactions.

### 2.4. Genome Survey of bsh Genes in Lactobacillaceae Species

Of 194 *Lactobacillaceae* species in the database of the National Center for Biotechnology Information (NCBI), 158 species had records of whole genome data by the end of year 2017. A total of 451 genomes from 158 *Lactobacillaceae* species were obtained to search for *bsh* genes. As a result, 551 BSH-homologous proteins were identified from 107 species. *bsh*-homologous genes were absent in 51 species. Among those species abundant in BSH-homologous proteins, which accounted for almost one-third of the species surveyed, *Lactobacillus plantarum*, *Lactobacillus oris* and *Lactobacillus brevis* are known as BSH-rich species, and eight BSH-rich species with more than three newly detected *bsh* genes were discovered, including *Lactobacillus acidifarinae*, *Lactobacillus antri*, *Lactobacillus farraginis*, *Lactobacillus odoratitofui*, *Lactobacillus rapi*, *Lactobacillus spicheri*, *Lactobacillus zymae* and *Lactobacillus parabuchneri*.

**Figure 4:**
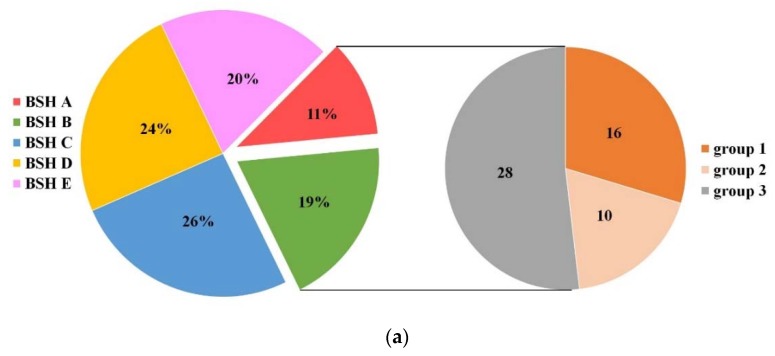

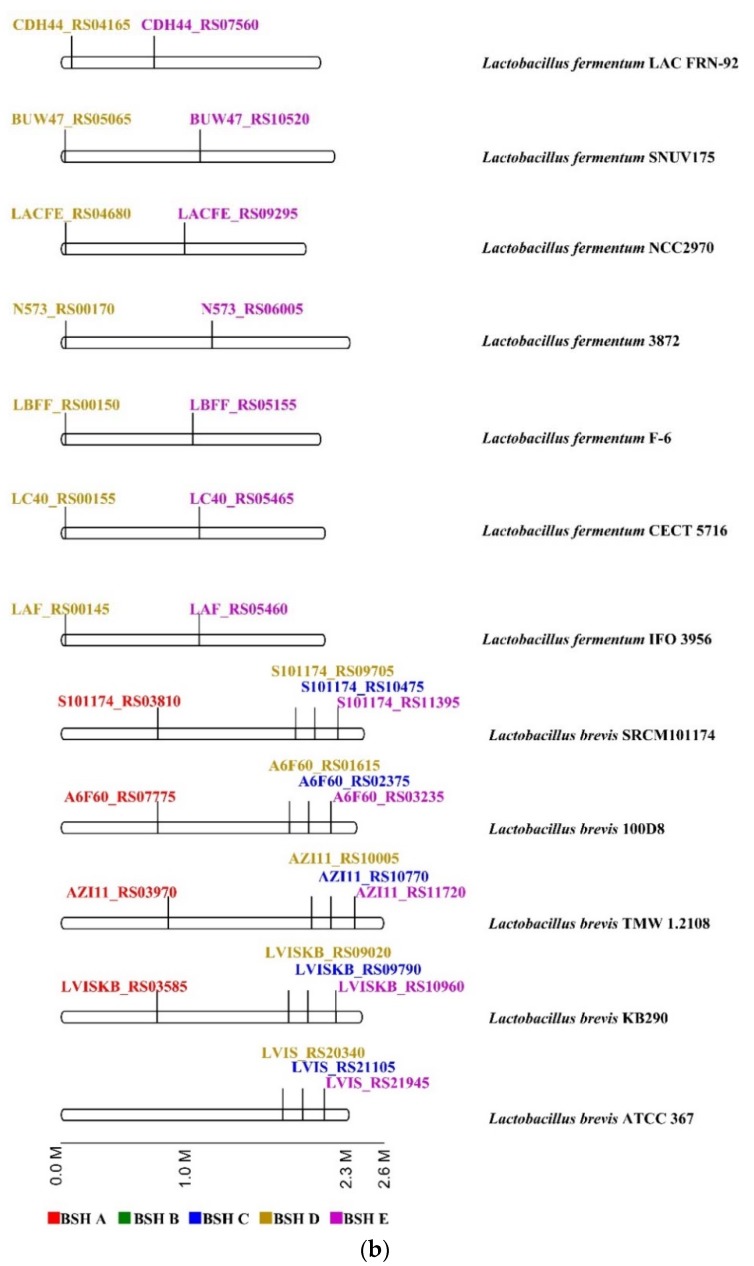
Distribution of BSH subtypes in 107 *Lactobacillaceae* species. (**a**) Proportion of each BSH subtype in 107 species. 54 species carrying BSH A or BSH B subtypes accounted for 30% in total. Group 1 consists of 16 species which have been verified for BSH activity, and is included in BSH–SDL, and the other 38 species in group 2 (10 potential cholesterol-lowering bacteria) and group 3 (28 species without related investigation) are predicted to be BSH-active based on this study; (**b**) genomic location of *bsh* in *Lactobacillus fermentum* and *Lactobacillus brevis*. Those colors marked on BSH subtypes (**a**) and gene ID (**b**) indicate their corresponding colors on BSH subtypes in the phylogenetic tree of Figure 2.

Homology searches identified the BSH subtypes of 551 BSH homologous proteins via their matched protein sequences in BSH–SDL. BSH proteins of the BSH C subtype were the most abundant BSHs in *Lactobacillaceae* species, while BSH A had the fewest (Figure 4a, Table S3). In addition, BSH A and BSH B proteins were identified in 54 species, and apart from the 16 species in BSH–SDL that exhibited BSH activity, 38 species were predicted to have potentially high BSH activity based on the results of phylogenetic and catalytic-residue analysis in this study. Interestingly, reported BSH activity studies suggest that 10 species of these 38 species are capable of hydrolyzing BAs or have resistance to bile, including *Lactobacillus agilis*, *Lactobacillus animalis*, *Lactobacillus gallinarum*, *Lactobacillus helveticus*, *Lactobacillus ingluviei*, *Lactobacillus intestinalis*, *Lactobacillus kefiranofaciens*, *Lactobacillus panis*, *Lactobacillus taiwanensis* and *Lactobacillus vaginalis*, and the other 28 species do not yet have relevant research conducted on them, including *Lactobacillus lindneri*, *Lactobacillus hominis*, *Lactobacillus harbinensis*, *Lactobacillus ruminis*, *Lactobacillus intestinalis* and so on (Figure 4a, Table S4). In Table 1, several representative BSH-active strains with diverse BSH subtypes are listed. It is demonstrated that our predictive results of BSH subtypes identified in their whole genome are consistent with their biological activities verified by previous studies.

Moreover, the complete genomes of species carrying multiple *bsh* copies were employed to investigate the genomic location of *bsh* genes. It was revealed that various BSH subtype genes of one strain are not clustered. Concurrently, the locations of one specific *bsh* gene subtype in different strains of the same species are relatively fixed (Figure 4b, Figure S1). For instance, it is fully illustrated that two different BSH subtype genes of *Lactobacillous fermentum* are distributed in the different positions of seven complete genomes of *Lactobacillous fermentum* strains, and four BSH subtypes of *Lactobacillous brevis* are located, in a relatively fixed positioning pattern, at five genomes of strains in this species (Figure 4b). The same results were also exhibited in other *Lactobacillaceae* species and strains (Table S5), while different species showed different *bsh* distribution patterns. Therefore, it is demonstrated that *bsh* genes have significant variation among species, while they are comparatively conserved within strains of one species.

## 3. Discussion

BSHs have attracted much attention because of their vital role in cholesterol metabolism, which makes them a common indicator for screening probiotics. Although several BSH studies have been investigated in a number of strains, in-depth exploration in *Lactobacillaceae* is limited. Here, a comprehensive molecular investigation into BSHs in *Lactobacillaceae* was performed. In this study, BSHs of *Lactobacillaceae* were divided into five subtypes based on their phylogenetic relationship, and BSH A and BSH B are two subtypes possessing high hydrolysis activity. Six catalytic residues were compared among 60 proteins of five BSH subtypes. It was observed that the diversity of amino acid residues in site 82 was a key factor to distinguish BSH subtypes. Identification of BSH-homologous proteins in 451 genomes from 158 *Lactobacillaceae* species revealed that 54 species were predicted to be BSH-active bacteria, as *bsh* genes of BSH A or BSH B subtype exist in their genomes. While 28 species have not been reported with any BSH-related research, they are regarded as potential bacterial strains with BSH activity. For example, *Lactobacillus intestinalis* was isolated from the intestinal tract and it was reported that a high-fat diet changed its abundance in the gastrointestinal tract of rats [32]; however, detailed BSH verification and other characteristics were unclear. Therefore, these 28 species could be valuable resources to discover novel probiotics with higher BSH activity, and this study provides new clues to related intestinal microbiology studies.

Some *Lactobacillaceae* species carrying multiple BSH copies have been reported in previous studies, but their functional annotations and nomenclature of BSH subtypes were not consistent among species. Therefore, a standardized BSH classification, which is based on phylogenetic analysis of BSH proteins on BSH–SDL, is provided in this study. Newly discovered BSH protein sequences could distinguish their subtypes by referring to five BSH subtypes proposed in our analysis results. It would be helpful for later researchers to recognize BSH protein sequences comprehensively and to share their research results more easily. This high-throughput approach bridges the gap between traditional isolation analysis and bioinformatics.

In the KEGG database, 34 *Lactobacillaceae* species carrying *bsh* genes were recorded, while in this study, BSH-homologous protein sequences have been identified in 107 *Lactobacillaceae* species. Many studies have demonstrated that BSH A and BSH B subtype proteins had greater BSH activity. Therefore, the combination of previous observations and BSH phylogenetic relationship analysis results could be employed to infer whether a strain expressing BSH-homologous protein exhibits BSH-active ability or not. In our results, *Lactobacillus* sp. wk88, *Lactobacillus lindneri*, *Lactobacillus heilongjiangensis*, *Lactobacillus ginsenosidimutans* and *Lactobacillus ruminis* are considered as potential BSH-active probiotics (Figure 2). In addition, it is practicable to identify the exact contributory molecules for cholesterol-lowering effects in multiple BSH proteins of *Lactobacillus mucosae* LM1 (LBLM1_10135). For *Lactobacillus amylovorus* GRL 1112 and *Lactobacillus gasseri* ATCC 33323, two BSH proteins in each are predicted to be BSH-active in hydrolyzing BAs.

Although amino acid residues of site 82 in BSH proteins are predicted to be closely related to BSH activity in this study, site-directed mutagenesis of *bsh* genes on this site was rarely found to provide direct proof of this conclusion. Experimental data from *Lactobacillus salivarius* [33] seemed to support this point indirectly, as it showed that the BSH mutation of Asn82 into Ala82 significantly decreased its capability of catalyzing TCA, while other mutant amino acids in other sites also influenced the BSH activity. However, catalytic residues of Cys2, Arg18, Asp21, Asn175 and Arg228 are highly conserved, and it would be easier to distinguish the biological effects of amino acid residues in site 82 and to explore their relationship with BSH-active subtypes. Our analysis provides new site information and sequence characteristics for further BSH exploration.

## 4. Materials and Methods

### 4.1. Establishment of BSH–SDL

All of the bile salt hydrolase (choloylglycine hydrolase, ko: K01442) protein sequences from *Lactobacillaceae*, which have records in the KEGG database (http://www.kegg.jp/dbget-bin/www_bget?ko:K01442), were collected in our local database. Those redundant protein sequences were removed by mutual BLASTP alignment (e-value: 1e-10), and sequences with more than 90% identity were deleted. A nonredundant *bsh*-seed database BSH–SDL was built for further analysis.

### 4.2. Identification of BSH in Lactobacillaceae

A total of 194 *Lactobacillaceae* species were recorded in NCBI by the end of August 2017, and 158 species have genome data, thus a total of 451 whole-genome-derived protein sequence datasets of *Lactobacillaceae* strains from 158 *Lactobacillaceae* species were retrieved from the NCBI RefSeq database (https://www.ncbi.nlm.nih.gov/) (Table S6). Subsequently, protein sequences in BSH–SDL were employed to search against 451 protein datasets using BLASTP (e-value: 1e-5), in order to identify homologous BSH sequences in different strains. Those hits with identity less than 60% were removed, and the best hit was retained for the following analysis using an in-house pipeline.

### 4.3. Genomic Location of bsh

The whole complete genomes from 451 *Lactobacillaceae* strains were selected to investigate the genomic location of *bsh* genes. To normalize the starting position of the 451 genomes, the *dnaA* gene was regarded as the initial gene. The gene location had been readjusted by an in-house Python script if *dnaA* was not the initial gene in the records of NCBI. The presentation of genomic location of *bsh* was accomplished by TBtools software (version 0.49991, CJ-Chen, Guangzhou, China) [34].

### 4.4. Sequence Alignment and Phylogenetic Analysis

The protein sequences in BSH–SDL were used for the construction of the phylogenetic tree. At first, MUSCLE (version 3.7, CV, Mill Valley, CA, USA) [35] was employed to perform multiple sequence alignment with default parameters. Sequence analysis was further shown by TeXshade (version 1.25, Eric Beitz, Tübingen, Germany) [36]. Then, the phylogenetic tree was constructed by PhyML (version 3.1, LIRMM, Université de Montpellier, Montpellier, France) [37] with best-fit protein evolution model (WAG+I+G+F), which was confirmed by Prottest 3 software (version 3.4.2, University of Vigo, Vigo, Spain) [38], and with a bootstrapping value of 1000. Final tree visualization was processed by iToL (version 4.2, biobyte solutions GmbH, Heidelberg, Germany) [39].

## 5. Conclusions

In this study, the genetic basis of *bsh* genes in *Lactobacillaceae* species has been explored systematically. Phylogenetic analysis indicates that BSH can be classified into five subtypes, which provides a general principle for future nomenclature of BSH protein. Further multiple sequence alignment demonstrates distinct characteristics of BSH A, B, C, D and E in *Lactobacillaceae*, particularly in site 82. We point out that Gln82 and Asn82 are two critical amino acids distinguishing BSH-active subtype proteins from other BSHs with lower BA hydrolysis activity. In addition, a large-scale high-throughput identification of *bsh* genes in genomes of all available *Lactobacillaceae* species provides a novel approach to select potential BSH-active candidate probiotics for cholesterol-lowering effects. Genome location of *bsh* in various species also indicates its conservation and diversity in *Lactobacillaceae*. These findings provide novel insights into BSH, and valuable resources for further research on cholesterol-lowering probiotics.

## Figures and Tables

**Table 1:** Biological activity of representative predicted BSH-active species with diverse BSH subtypes.

Species	BSH Subtypes					Tolerable Conditions		Substrate Specificities	Strains
	A	B	C	D	E	Bile Salt (%)	pH		
*Lactobacillus plantarum*		1	2		1	1.0	2.0	GCA	ST-III [20]
		1	2		1	-	-	GCA	WCFS1 [19]
		1	2		1	0.3	2.0	-	LZ95 [26]
		1	1		1	1.0	2.0	-	NCU116 [27]
*Lactobacillus acidophilus*		2				0.2	-	TCA, GCA, TCDCA	NCFM [22]
		2				0.3	2.0	-	DSM 20079 [28]
*Lactobacillus johnsonii*		2				0.5	-	-	PF01 [29]
		2				0.5	2.0	GCDCA, TCDCA	La1 (also known as NCC533) [30]
*Lactobacillus rhamnosus*	1					2.0	2.5	-	GG (ATCC 53103) [31]

## Supplementary Materials

Supplementary materials can be found at http://www.mdpi.com/1420-3049/23/5/1157/s1. Figure S1: Genome location of *bsh* genes in 13 *Lactobacillaceae* species; Table S1: Detailed information about protein sequences in BSH–SDL; Table S2: Statistics of six catalytic residues of BSH subtypes from all BSH protein sequences in BSH–SDL; Table S3: Statistics of mapped BSH subtypes in 158 species; Table S4: Statistics of mapped information about BSH A and BSH B in 54 species; Table S5: The genomic location of *bsh* in representative *Lactobacillaceae* species; Table S6: The NCBI ftp list of 451 *Lactobacillaceae* genomes.

## Abbreviations

CVD	cardiovascular disease
TC	total cholesterol
LDL-C	low-density lipoprotein cholesterol
BAs	bile acids
CA	cholic acid
CDCA	chenodeoxycholic acid
TCA	taurocholic acid
TCDCA	taurochenodeoxycholic acid
GCA	glycocholic acid
GCDCA	glycochenodeoxycholic acid
BSH	bile salt hydrolase
BSH–SDL	bile salt hydrolase seed database of *Lactobacillaceae*
FXR	farnesoid X receptor
GPBAR1/TGR5	G-protein-coupled bile acid receptor 1
