# Supplementary figures and images for: A Comprehensive Genome Survey Provides Novel Insights into Bile Salt Hydrolase (BSH) in Lactobacillaceae
Source: Molecules. 2018 May 11;23(5):1157. doi: 10.3390/molecules23051157 (PMC6100381; doi:10.3390/molecules23051157)

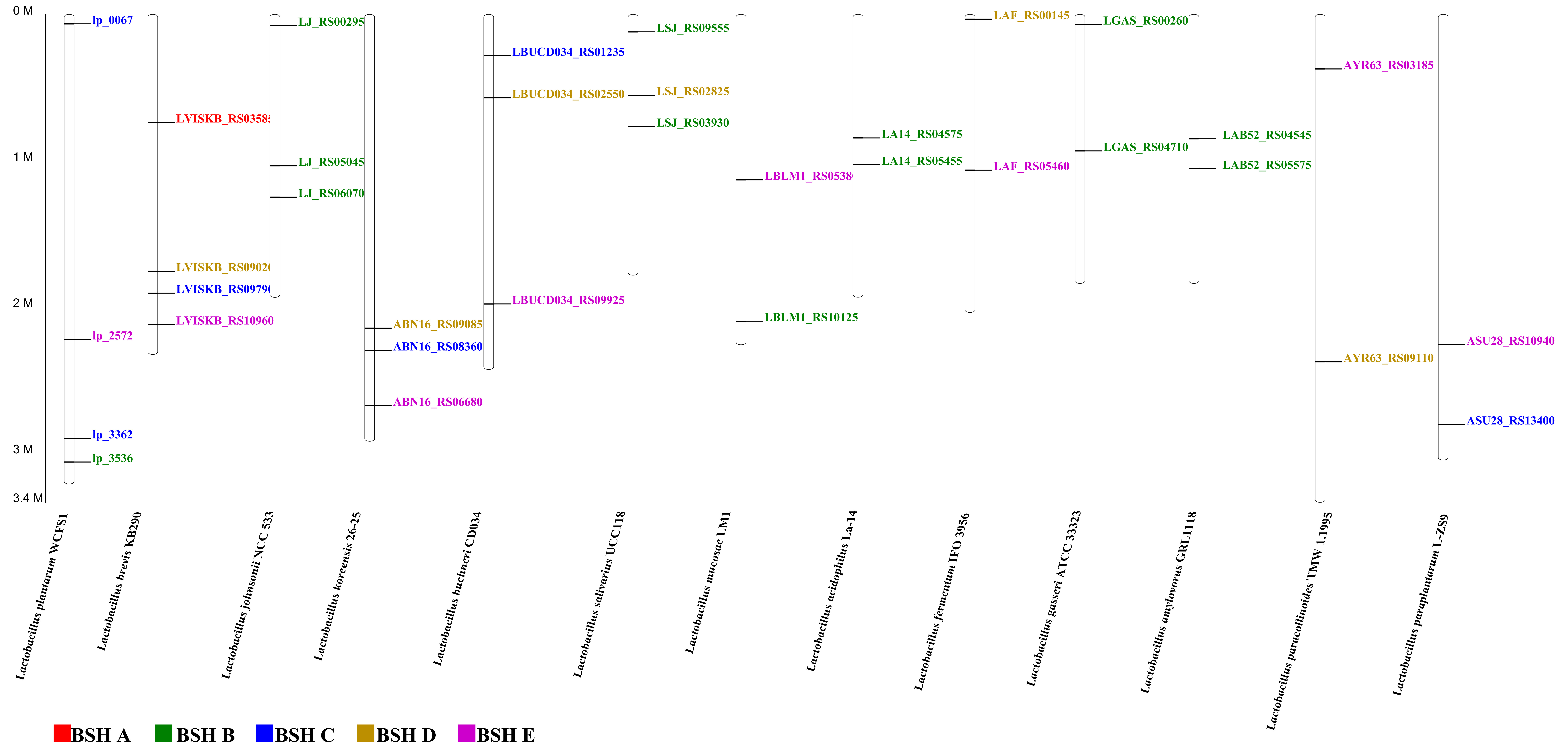

Supplement: Supplementary file 1 [file molecules-23-01157-s001.zip › Supplementary/Figure S1.jpeg]
